# Supplementary material for: Transcriptomic Insights into the Effects of Inoculation Density in Areca catechu Tissue Culture
Source: Plants (Basel). 2025 Oct 4;14(19):3073. doi: 10.3390/plants14193073 (PMC12526192; doi:10.3390/plants14193073)
Supplement: Supplementary file 1 [file plants-14-03073-s001.zip › plants-3878270-supplementary.pdf]

Supplementary Table S1: A table listing the exact numbers of up- and down-regulated DEGs and TFs for CK9 vs. GL9 and CK12 vs. GL12 etc.

Contrast and combination	Number of upregulated genes	Number of downregulated genes
CK9vsGL9	143	144
CK12vsGL12	271	574
CK15vsGL15	284	879
CK18vsGL18	132	416
CK21vsGL21	367	452

Contrast and combination	Number of upregulated transcription factors	Number of downregulated transcription factors
CK9vsGL9	2	1
CK12vsGL12	14	15
CK15vsGL15	32	68
CK18vsGL18	6	14
CK21vsGL21	18	13
